# Development of a Versatile Toolbox for Genetic Manipulation of *Sporothrix brasiliensis*

**DOI:** 10.1128/spectrum.04564-22

**Published:** 2023-02-27

**Authors:** Matheus Tavares, Jorge Carlos Sousa-Filho, Ian Alves Machado, Relber Aguiar Gonçales, Daniela Antunes, Ana Mendes-Frias, Ricardo Silvestre, Agostinho Carvalho, Egídio Torrado, Cristina Cunha, Fernando Rodrigues

**Affiliations:** a Life and Health Sciences Research Institute (ICVS), School of Medicine, University of Minho, Braga, Portugal; b ICVS/3B's-PT Government Associate Laboratory, Guimarães/Braga, Portugal; Universidade de Sao Paulo

**Keywords:** *Sporothrix brasiliensis*, *Agrobacterium tumefaciens*, ATMT, genetic toolbox, sporotrichosis, GFP, mCherry

## Abstract

*Sporothrix brasiliensis* has emerged as the most virulent species in the *Sporothrix schenckii* complex, accounting for sporotrichosis. Albeit the new insights into the understanding of host-pathogen interactions and comparative genomics of this fungi, the lack of genetic tools has hindered significant advances in this field of research. Here, we established an Agrobacterium tumefaciens*-*mediated transformation (ATMT) system to transform different strains of *S. brasiliensis*. We report parameters that account for a transformation efficiency of 3,179 ± 1,171 transformants/co-cultivation, which include the use of A. tumefaciens AGL-1 in a 2:1 ratio (bacteria:fungi) during 72 h at 26°C. Our data show that a single-copy transgene is transferred to *S. brasiliensis* that is mitotically stable in 99% of cells after 10 generations without selective pressure. In addition, we created a plasmid toolkit that allows the establishment of fusion proteins of any *S. brasiliensis* gene of interest with sGFP or mCherry under the control of the GAPDH or H2A endogenous promoters. These modules allow different levels of expression of the desired fusion. Moreover, we successfully targeted these fluorescent proteins to the nucleus and used fluorescence-tagged strains to assess phagocytosis. Overall, our data show that the ATMT system is an easy-to-use and efficient genetic toolbox for studies on recombinant expression and gene function in *S. brasiliensis*.

**IMPORTANCE** Sporotrichosis is the most prevalent subcutaneous mycosis worldwide and has recently become a public health concern. Although immunocompetent hosts are also prone to sporotrichosis, immunodeficient hosts often develop a more severe and disseminated form of disease. To date, the Rio de Janeiro state in Brazil is the most significant feline zoonotic transmission epicenter in the world, with more than 4,000 human and feline diagnosed cases. Cats play an essential role in the *S. brasiliensis* infection due to their high susceptibility and transmissibility to other felines and humans. *S. brasiliensis* is the most virulent etiological agent of sporotrichosis, causing the most severe clinical manifestations. Despite the increasing incidence of sporotrichosis, the identification of virulence traits important for disease establishment, development, and severity has been lacking. In this work, we established an efficient genetic toolbox to manipulate *S. brasiliensis* that will guide future studies to define new virulence mechanisms and a better understanding of host-pathogen interactions from a molecular perspective.

## INTRODUCTION

Sporotrichosis is the world's most prevalent and distributed subcutaneous mycosis caused by the traumatic inoculation of the dimorphic fungus *Sporothrix schenckii* complex, including *S. schenckii s str*, *S. globosa*, *S. brasiliensis*, and *S. luriei* (1). *S. schenckii s str* and *S. brasiliensis* are the most virulent species and the main etiological agents of sporotrichosis. However, while infections caused *by S. schenckii s str* are globally endemic, those associated with *S. brasiliensis* are localized in South America, with Brazil being a hyperendemic country (1). Recent data show that the most severe and lethal forms of *S. brasiliensis* infections in cats lead to massive transmissions of this pathogen to humans (zoonotic route) (2). While sporotrichosis has been successfully treated with standardized antifungal strategies, the drastic increase in its prevalence has placed sporotrichosis as an emerging health problem that will potentially worsen in the future (1). Therefore, it is important to increase our understanding of the pathogenic mechanisms of *S. brasiliensis*. Indeed, despite the well-defined role of dimorphism, thermotolerance, and melanin, the virulence characteristics involved in the establishment, development, and severity of sporotrichosis are still unclear (3). To overcome this knowledge gap, a new repertoire of forward and reverse genetic manipulation tools to facilitate the identification and characterization of genes that govern features of pathogenicity and host specificity are urgently required (4).

Among many fungal genetic transformation systems, the Agrobacterium tumefaciens-mediated transformation (ATMT) system has been previously applied to other pathogenic fungi, particularly dimorphic, as it is highly efficient and easy to operate (5–8). Moreover, this system delivers genetically stable transformants with a single copy of T-DNA insertion in the genomes (9). As such, ATMT offers an efficient tool for random insertional mutagenesis of DNA into intact cells, including yeast, conidia, and mycelium (10). To date, different ATMT transformation protocols have been developed for fungi. These protocols are based on mixing transformation-competent A. tumefaciens cells, which carry an engineered plasmid containing a fungus-specific selectable marker with fungal host cells. However, the efficiency of these systems depends on several factors, namely, the A. tumefaciens strain, the morphological state of the fungal cell (conidia, yeast, mycelium), and the time and temperature of co-cultivation, acetosyringone being an essential inducer of virulence genes during co-cultivation, thus facilitating the transfer of the T-DNA region (11). Although ATMT has been optimized for several species, this system has not been developed for *S. brasiliensis*.

Herein, we established and optimized an ATMT system to transform efficiently different strains of *S. brasiliensis*. We report the use of this system to drive the expression of red and green fluorescent proteins in *S. brasiliensis*, under the control of the endogenous promoters of glyceraldehyde-3-phosphate dehydrogenase and histone H2A genes. The generated *S. brasiliensis* fluorescent-tagged strains were then used in proof-of-concept immunological studies. Overall, we showed an efficient genetic toolbox to construct large-scale transformant libraries for *S. brasiliensis*, which can help in providing a better understanding of host-*Sporothrix* interactions and pathogenic mechanisms that may open new insights to explore more effective disease control strategies for sporotrichosis.

## RESULTS

### Agrobacterium tumefaciens-mediated transformation protocol for *Sporothrix brasiliensis*.

**FIG 1:**
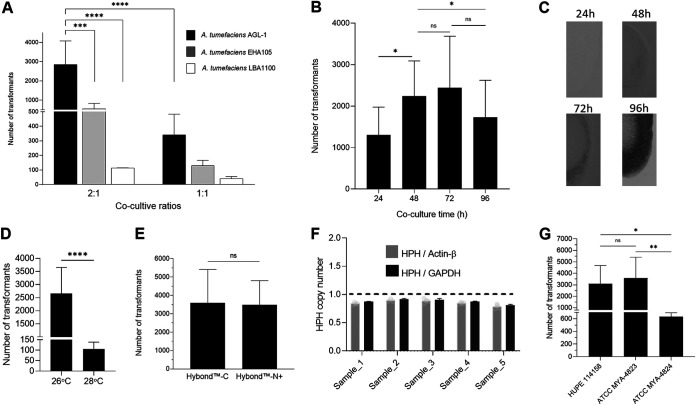
Factors affecting the efficiency of ATMT of *S. brasiliensis*. (A) Effect of A. tumefaciens strains (LBA11000; EHA 105 and AGL1; harboring binary vector pUR5750, where HPH expression is driven by the promoter of glyceraldehyde-3-phosphate dehydrogenase from Aspergillus nidulans) and the ratio of *Agrobacterium:Sporothrix* on transformation efficiency. The growing colonies on the hygromycin medium were counted as transformants. The number of transformants ± SD per condition is represented as bars. Two-way ANOVA determined statistically significant data by Tukey's multiple-comparison tests (****, *P* < 0.0001; *n* ≥ 4). (B) Effect of length of duration of co-cultivation on transformation efficiency. Mean values of the number of transformants ± SD per time are represented. One-way ANOVA determined statistically significant data by Tukey's multiple-comparison tests (*, *P* < 0.05; *n* = 6). (C) Filter pieces transferred to recover medium after different co-cultivation times. (D) Effects of co-cultivation temperature in *S. brasiliensis* ATMT efficiency. Bars depict the mean ± SD number of transformants per condition. The Student's *t* test determined statistically significant data (****, *P* < 0,0001; *n* ≥ 10). (E) Effect on *S. brasiliensis* ATMT efficiency between the use of Hybond-C or Hybond-N^+^ in co-cultivation. Bars depict the mean ± SD number of transformants per condition. No statistical difference was observed between the membranes (*n* = 4). (F) T-DNA copy number of *S. brasiliensis* transformants, based on Actin-β and glyceraldehyde-3-phosphate dehydrogenase gene (GAPDH) as a single-copy reference gene and hygromycin phosphotransferase (HPH) as a proxy for T-DNA insertion event. (G) The effect of the strain *S. brasiliensis* on transformation efficiency. Bars show the mean number of transformants ± SD per condition. One-Way ANOVA determined statistically significant data by Tukey's multiple-comparison tests. (****, *P* < 0.0001; ***, *P* < 0.001; *, *P* < 0.05; *n* ≥ 4).

To establish an efficient method for gene transformation in *S. brasiliensis*, an absolute requirement for genetic manipulation, we took advantage of the highly recognized ATMT systems established for other dimorphic fungi (5–8). To apply this system to *S. brasiliensis*, we determined the optimal conditions of ATMT on transformation efficiency, including (i) A. tumefaciens strain, (ii) co-cultivation ratios (bacteria:fungi), (iii) co-cultivation time, (iv) co-cultivation temperature, and (v) co-cultivation filter matrix. We began by transforming *S. brasiliensis* MYA-4823 with different A. tumefaciens strains (AGL-1, EHA105, and LBA1100) harboring the pUR5750 plasmid carrying HPH as a selection marker for hygromycin B. We found that A. tumefaciens AGL-1 is the most virulent strain for *S. brasiliensis* at a 2:1 ratio of coculture (bacteria/fungus) (Fig. 1A).

We next determined the coculture time that yielded the highest number of transformants. We used A. tumefaciens AGL-1 and *S. brasiliensis* at a 2:1 ratio and found that after 48 h to 72 h of coculture, the number of transformants was higher than those obtained after 24 h or 96 h of coculture (Fig. 1B). However, upon 96 h of culture, *S. brasiliensis* formed a biofilm-like structure that impaired the removal of cells from the sterile membrane being responsible for the reduced number of transformants (Fig. 1C). We then tested the impact of coculture temperature in transformation efficiency and found that a higher number of transformants was obtained at 26°C (Fig. 1D). No effects on transformation efficiency were found using either Hybond-N+ or Hybond-C membranes during coculture (Fig. 1E). These data show that the use of ATMT with A. tumefaciens AGL-1 at a 2:1 ratio with *S. brasiliensis* for 48 h to 72h at 26°C is ideal for the transformation of *S. brasiliensis*. For selection purposes the cell recovery time/*Agrobacterium* killing (6 h) can be skipped by directly platting transformed cells into selective medium containing cefotaxime and hygromycin B without effecting the transformation efficiency (data not shown).

With the established conditions, we evaluated the number of T-DNA ectopic integrations in the genome of *S. brasiliensis*. To this end, we randomly selected five transformants resistant to hygromycin B, and their T-DNA copy number was determined by real-time PCR analyses using actin-beta (SPBR_00592) and glyceraldehyde-3-phosphate dehydrogenase (GAPDH, SPBR_04305) as two reference genes of a known single copy per cell haploid genome (12–15). Our data revealed that all analyzed transformants have a single copy of T-DNA inserted in their genomes (Fig. 1F). Overall, these data show that the ATMT system is an efficient method to transform *S. brasiliensis.* As a proof of concept, we successfully applied this system to other *S. brasiliensis* strains (Fig. 1G).

### Fluorescent-tagged *S. brasiliensis* is suitable for phagocytosis assays using flow cytometry.

**FIG 2:**
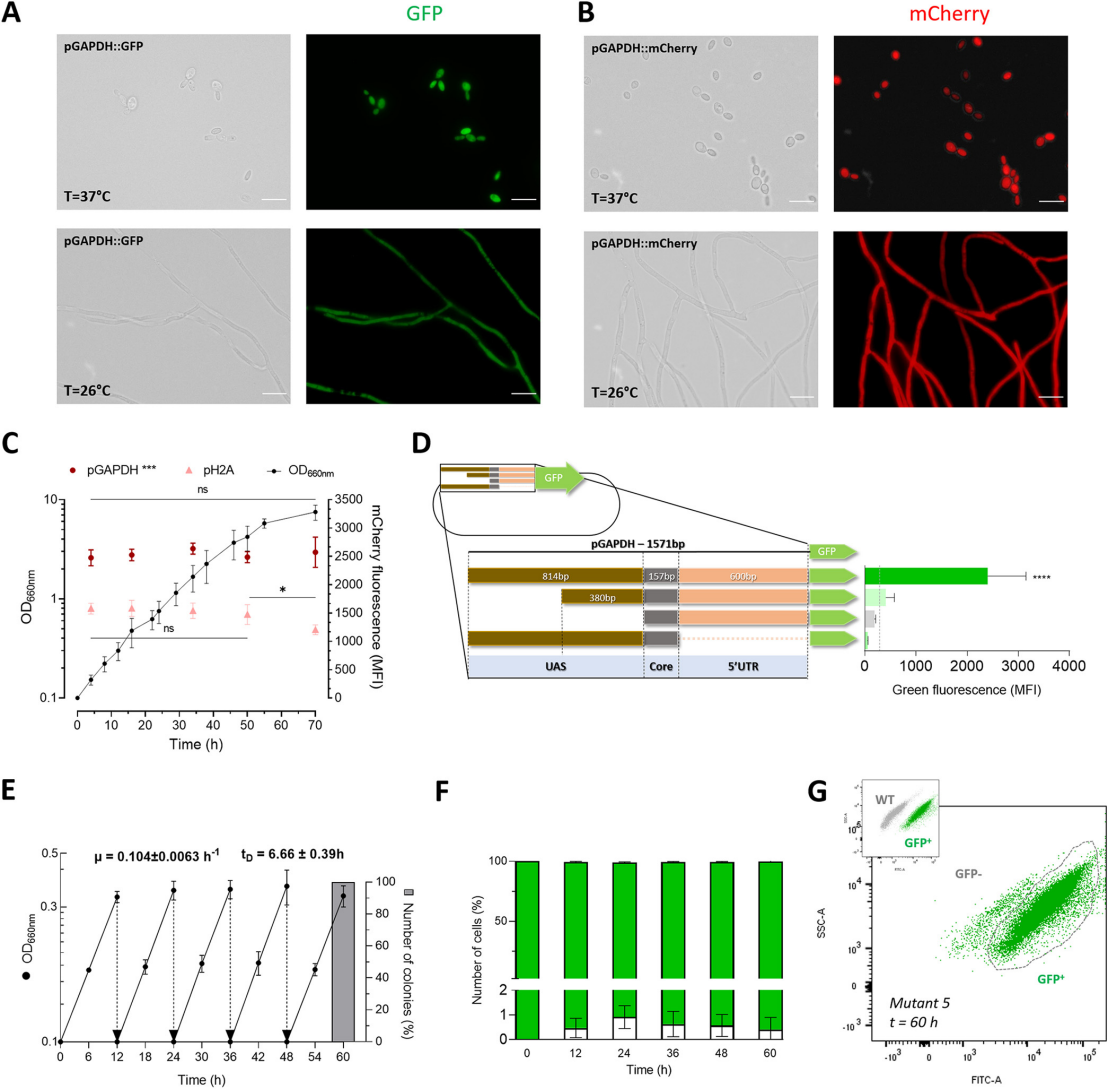
Mitotic stability and promoter evaluation of different *S. brasiliensis* transformants through sGFP and mCherry gene expression. The scale bar equals 10 μm for all images. (A) Fluorescence microscopic analysis of the green fluorescent *S. brasiliensis* tagged strain, expressing sGFP during yeast and mycelium phases through the Bright field and FITC channels. (B) Fluorescence microscopic analysis of the red fluorescent *S. brasiliensis* tagged strain, expressing mCherry in the cytoplasm during both yeast and mycelium phases through the Bright field and TRITC channels. (C) Differences between pGAPDH and pH2A promoters in *S. brasiliensis* mCherry expression, along a batch growth culture. Data depicts the mean of OD_660nm_ ± SD, illustrated as dots, correlated with the mean of MFI expressed by pGAPDH and pH2A promoters through time, demonstrated as dots and triangles, respectively. (***, *P* = 0,0001; *, *P* < 0.5; *n* = 6). Two-way ANOVA determined statistically significant data by Sidak's multiple-comparison test (***, *P* < 0.001; *, *P* < 0.5; *n* = 5). (D) The difference in sGFP expression driven by different lengths of the GAPDH promoter. The GAPDH is a TATA less promoter, and region of 1,571 bp upstream of the start codon was considered full-length promoter. Successive deletion of nucleotides −1,571 to −1,137, −1,571 to −757, and −584 to −1 from the starting codon were used to evaluate the presence of essential acting elements related to transcription activity located within these regions. The wild-type *S. brasiliensis* autofluorescence threshold is indicated by the traced line. Bars depict the mean ± SD number of MFI per condition, namely, *S. brasiliensis* tagged strains with the expression of sGFP through the activity of different lengths of the GAPDH promoter as schematically represented. One-way ANOVA determined statistically significant data by Dunnett's multiple-comparison test (****, *P* < 0.0001; *n* = 5). (E) Growth profile of five randomly selected tag-strain colonies, in a nonselective medium, with five consecutive dilutions. The data represent means of OD_660nm_ ± SD after five restreaks of 12 h of incubation (*n* = 5), with the exponential growth rate (μ) and duplication time (T_D_). The gray column represents the percentage of resistant colonies to hygromycin B after 60 h from 100 colonies from 10 randomly selected clones. (F) Percentage of cells expressing sGFP across all five consecutive cultivations, each with a twelve-hour growth period in a nonselective medium. Bars depict the mean MFI ± SD percentage of negative cells per restreak (*n* = 5). (G) Dot plot of side scatters (SSC-A LOG) versus green fluorescence intensity (FITC-A). The upper left inset plot shows the differences in green fluorescence intensity between sGFP-tagged strains and the wild type. These differences were used to define the gating strategy. The dot plot represents the data obtained after 60h of growth in a nonselective medium.

The developed ATMT is crucial to increase the repertoire of molecular tools available for *S. brasiliensis* research. Therefore, we used this methodology to evaluate the application of green fluorescent protein enhanced synthetic variant sGFP (S65T) and the improved monomeric red fluorescent protein mCherry (16) in this pathogen for immunological and cell biology studies. To drive the expression of these fluorescent markers and ensure constitutive expression, we cloned around 1,500 nucleotides upstream from the starting codon of GAPDH or the histone H2A genes. Our data showed that both promoters led to a homogeneous green or red fluorescence distribution over the cell, indicative of a cytosolic localization in yeast and mycelium (Fig. 2A and B; data for pH2A not shown). On the other hand, flow cytometry analysis of *S. brasiliensis* transformants revealed that the pGAPDH promoter induced strong fluorescence throughout the batch growth (Fig. 2C). On the other hand, pH2A led to a lower fluorescence that was constant only during the exponential growth phase, decreasing toward the stationary growth phase (Fig. 2C). These results are likely associated with the dependence of pH2A expression with the replicative status of the cell (17). We further attempted to reduce the size of pGAPDH to facilitate cloning. However, we found a drastic reduction of GFP expression in all constructs tested, as determined by mean fluorescence intensity (Fig. 2D).

Having established these new cell lines, we sought to evaluate the mitotic stability of the integrated T-DNA in the produced transformants. To this end, we analyzed the fluorescence intensity among wild-type and sGFP-positive clones in five randomly selected transformants. These clones were grown in batch under nonselective conditions (absence of hygromycin B pressure) for 12 h (~2 duplication times) for five consecutive periods (Fig. 2E). The green fluorescence was measured (from 30,000 cells) at the end of each batch growth, and compared with wild-type cells. These data showed that more than 99% of the analyzed cells maintained the green fluorescence at the initial level (Fig. 2F and G). To confirm the stability of the integration, we confirmed the hygromycin B resistance of one hundred isolated clones (from each 10 randomly selected transformants) after the five growth cycles described above (Fig. 2E). Taken together, these results strongly support the conclusion that *S. brasiliensis* transformants genomic insertion is mitotic stable.

Moreover, these strains did not show differences in terms of growth rate as well as dimorphic switch from yeast to mycelium following a decrease in temperature from 37°C to 26°C (data not shown). These results indicate that ATMT promotes a stable integration in *S. brasiliensis*, allowing the production of sGFP or mCherry fluorescent tagged strains.

**FIG 3:**
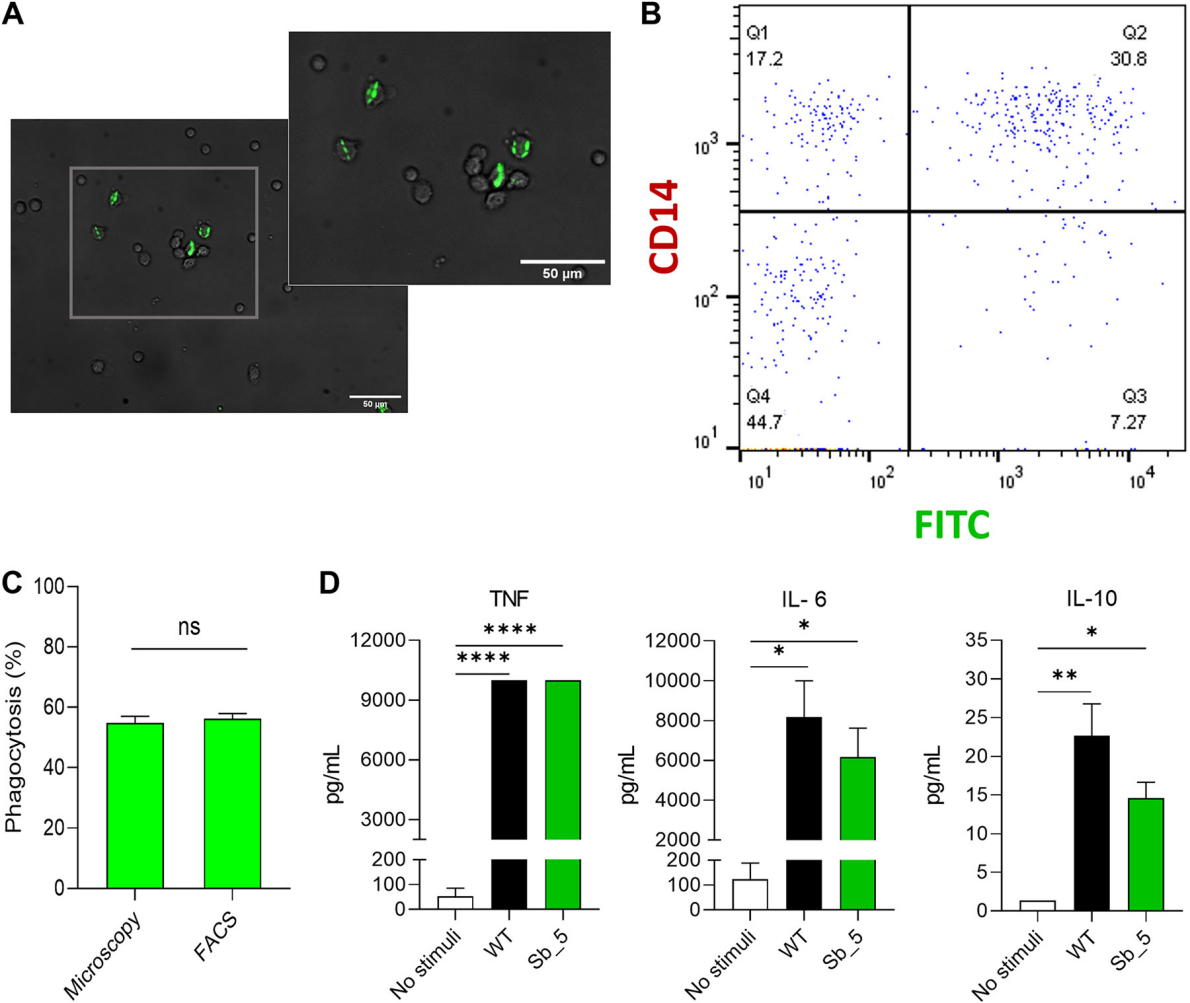
Green fluorescent *S. brasiliensis* tag strain used for phagocytosis assessment. (A) The green layer and bright fields were merged to visualize the green fluorescence of the sGFP *S. brasiliensis* tagged strain within CD14^+^ PBMCs. The scale bar equals 50 μm for both images. (B) Dot plot of BV650-A::CD14 versus the green fluorescence intensity (FITC-A). The subpopulation Q2 depicts monocytes (positive CD14) which phagocyted the sGFP *S. brasiliensis* tagged cells. (C) Phagocytosis was measured after infection with sGFP *S. brasiliensis* tagged cells at the MOI of 1:5 for 2h using flow cytometry and microscopic. Bars depict the percentage of phagocytosis ± SD (*n* ≥ 6). No statistical difference was observed. (D) Cytokine production of PBMCs infected with *S. brasiliensis* wild-type and GFP *S. brasiliensis* tagged strains. Bars depict the concentration of TNF, IL-6, and IL-10 after 24 h of stimulation of each strain. The data were expressed as mean ± SD. Statistically significant data were determined by one-way ANOVA by Tukey's multiple-comparison test (****, *P* < 0.0001; **, *P* < 0.01; *, *P* < 0.05; *n* ≥ 3).

The stable fluorescent phenotype of the obtained *S. brasiliensis* strains suggests that these may be a helpful tool for immunological studies, including fungal recognition and uptake by phagocytic cells, as established for other fungal pathogens (8, 18, 19). We used a sGFP-positive *S. brasiliensis* transformant in a phagocytosis assay with peripheral blood mononuclear cells (PBMCs) by microscopy and flow cytometry. Microscopic analysis revealed a clear and stable sGFP expression of *S. brasiliensis* inside the PBMCs, which was easily quantified. Accordingly, we found that 54.83% ± 5.39 of green fluorescent tagged *S. brasiliensis* yeast were phagocytosed by PBMCs, and flow cytometry analysis revealed a comparable value of 56.18% ± 4.97 (Fig. 3A to C). Similar results were obtained using human monocyte-derived macrophages (Fig. S1) and calcofluor-white labeled wild-type *S. brasiliensis* yeast cells (data now show). Furthermore, infection with isogenic yeast strains that express or not sGFP revealed no differences in cytokine production by PBMCs, namely, TNF, IL-6, and IL-10 (Fig. 3D), showing no deleterious effect of sGFP expression in immunological assays.

### A plasmid toolkit for flexible use of fluorescent protein fusions in *S. brasiliensis*.

**FIG 4:**
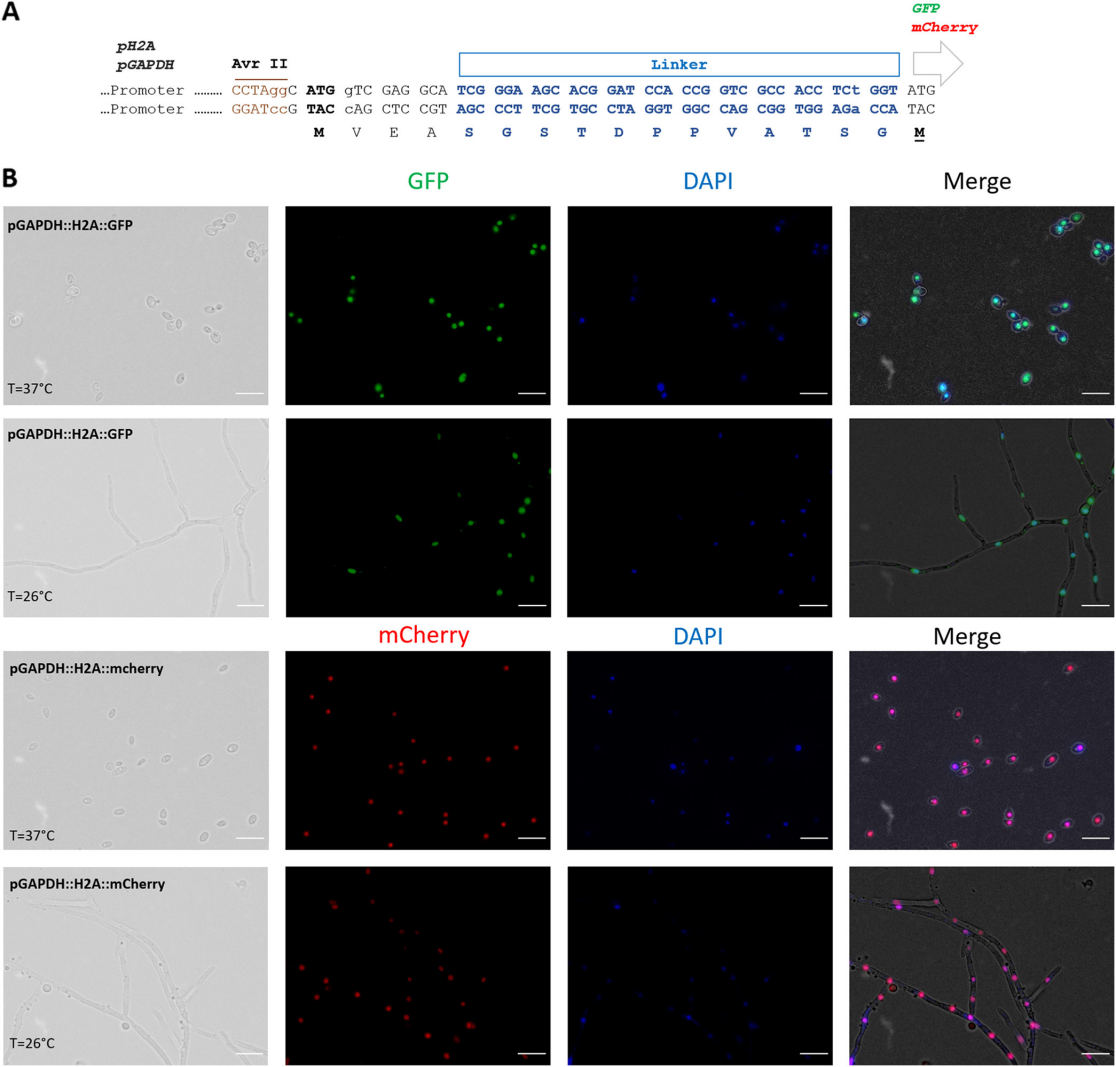
Production of fluorescent *S. brasiliensis* tagged strains with the pGAPDH promoter. (A) A representative scheme of the toolkit produced showing all the minimal modules composing the binary vectors, including the two promoters (pGAPDH and pH2A), the AvrII restriction cloning site, the translation starting codon, indicating the open reading frame, inert-linker, and sGFP or mCherry segments. (B) Fluorescence microscopic analysis of the *S. brasiliensis* strains, expressing sGFP or mCherry in-frame fused to the C-terminal of the H2A. The merged images show a high degree of co-localization between sGFP and mCherry expressions with the DAPI (4[prime],6-diamidino-2-phenylindole) nuclei staining during both yeast and mycelium phases. The scale bar equals 10 μm for all images.

We also constructed a plasmid toolkit for “easy-to-clone” and technically flexible use of green and red fluorescent proteins in *S. brasiliensis* of *in-frame* C-terminal fusions between genes of interest and sGFP or mCherry markers. We based our cloning strategy on the use of AvrII as the restriction enzyme to cut the plasmid backbone, and PCR for the amplification of the desired gene using long primers with a 5′-homologous sequence of 20 nt with the regions flanking the AvrII plasmid site for *in vivo* recombination cloning or *in vitro* assembly (20). To facilitate efficient gene cloning, we incorporated a unique nucleotide sequence around the AvrII site in both constructs harboring sGFP or mCherry, allowing us to test both markers with a single set of primers. The expression of the fusion protein is, in these systems, driven by the previously analyzed endogenous promoters, either pGAPDH or pH2A. In addition, we have included an inert and unstructured peptide to link the protein of interest and the fluorescent protein (21). Four different expression constructs were obtained (Fig. 4A).

To demonstrate the versatility of our expression plasmids, we next tested its application for *in vivo* analyses of the subcellular localizations of proteins using a common organelle marker gene, the histone H2A from *S. brasiliensis*. Our results demonstrate that an in-frame fusion of H2A with sGFP or mCherry relocalized the fluorescence in a subcellular compartment, which DAPI revealed to be the nucleus (Fig. 4B). The constructed fluorescent tagged strains showed a stable nuclear localization both in the yeast and mycelium morphological forms (Fig. 4B). We have also constructed an *S. brasiliensis* strain expressing both TURBOFP635/Katushka (22) and H2A::sGFP fluorescent proteins, revealing that strains with multiple fluorescent tags are feasible (Fig. S2).

## DISCUSSION

Herein, we optimized an ATMT system to produce a high number of transformants with a single copy integration. Our data showed that Agrobacterium tumefaciens ALG-1 is the most virulent strain to efficiently transform *S. brasiliensis*, compared to EHA105 and LBA1100 strains. The most efficient ratio of bacteria to fungi varies accordingly to each fungi species (23) together with the temperature of co-cultivation, because A. tumefaciens*'* T-DNA transfer functions are known to be thermolabile (24). The ratio 2:1 (bacteria:fungi) and the temperature of co-cultivation (26°C) also strongly impact transformation efficiency, The described method allows the production of an average of 3586 ± 1826 clones per transformation of single-copy T-DNA integration, which makes this system compatible with large-scale insertional mutagenesis for *S. brasiliensis* studies. This protocol was also suitable for application in different *S. brasiliensis* strains. Nonetheless, the transformation of *S. brasiliensis* ATCC MYA-4824 yielded a low number of clones, a fact that can be related with the strength of the glyceraldehyde-3-phosphate dehydrogenase promoter from Aspergillus nidulans that is driving the expression of the HPH gene, or with specific traits of the strain.

Genetic manipulation has revolutionized modern microbiology, with cell transformation being a critical tool to further our understanding of host-pathogen interaction. Indeed, implementing such tools had a major contribution in identifying virulence traits and cellular and metabolic remodeling that are critical for pathogenesis (25). This knowledge has been a steppingstone to developing novel targeted therapies.

In the past decade, the entire genome sequence of several species of the *Sporothrix schenckii* complex was a landmark in the ongoing quest to identify genes associated with host-pathogen interactions, dimorphism, or drug targets (26). Indeed, this knowledge has been critical to advance the field. For example, we recently used these data to elucidate the haploid state of the yeast cells of several species of *Sporothrix* (13), while others produced data sets on genes differentially expressed between yeast and mycelium forms of *S. schenckii s str* (27). Despite the functional insights which emerged from this differential expression analysis, the lack of genetic tools to modulate/disrupt gene expression has hampered the identification of molecular pathways associated with virulence, including those involved in dimorphism. In addition, in the *Sporothrix schenckii* complex, only the *S. schenckii s str* has a transformation system available (8, 10). As such, the development of molecular tools and the technical procedures to determine gene function, either by forward or reverse genetic techniques, in other species, including *S. brasiliensis*, was urgently needed. Therefore, the herein-developed transformation method will contribute to fostering research on *S. brasiliensis*.

Following the ATMT system optimization for research on *S. brasiliensis*, we also evaluated this system toward developing a gene expression system in this pathogen. To this end, we produced stable green and red fluorescent tagged *S. brasiliensis* strains and engineered a set of expression plasmids that allow the generation of red or green fusion proteins. These fluorescent reporter genes have been a crucial tool in cell biology, pathogenesis, and cell development biology studies. They provide a way to visualize, track, and quantify molecules and events in living cells with high spatial and temporal resolution (28). The set of plasmids we developed allows fluorescent fusions to any protein of interest in *S. brasiliensis* using Gibson technology or *in vivo* assembly cloning strategies (20).

Methodologically, any *S. brasiliensis* gene can be assembled into a vector’s backbone that has a common set of modules, such as endogenous promoters (from histone H2A or glyceraldehyde-3-phosphate dehydrogenase) and protein tags (sGFP or mCherry), at the AvrII unique restriction site to produce the final construct. We demonstrate the feasibility of this system by creating T-DNA constructs encoding sGFP or mCherry fluorescent proteins targeted to the cytosol or the nucleus. We believe that the availability of this toolkit will significantly help the future understanding of the biology/pathogenesis of this critical human/feline pathogen. In fact, the introduced vectors are handy tools for quick and efficient protein tagging in *S. brasiliensis*.

Due to the scarce knowledge of the transcriptome profile of *S. brasiliensis*, we evaluated the expression of sGFP and mCherry under the control of the promoters of histone H2A or GAPDH. We selected these two promoters. As in other cell systems, they are known to trigger high expression levels, being pGAPDH, and a constitutive promoter, whereas pH2A expression is mainly associated with the growth phase (17). Likewise, expression driven by pGAPDH was elevated throughout the growth batch phases. These data confirm the relevance of glyceraldehyde-3-phosphate dehydrogenase in the growth of *S. brasiliensis* in medium containing glucose as carbon and energy source. Comparatively, with pGAPDH, the promoter of H2A drives a lower expression that was even lower at the end of the exponential growth phase, aligned with the relevance of histone synthesis during cell division (17). However, the expression driven by pGAPDH can be highly dependent of glucose-containing growth media and consequently be reduced in certain media using alternative carbon sources; therefore, the promoter of H2A is of added value. Hence, this set of vectors allows the production of fusion proteins at higher or lower levels by selecting the promoter to be used, albeit the selection of the fluorescent protein. Interestingly, all our attempts to reduce the size of the pGAPDH to be used for cloning purposes had a strong negative impact on gene expression. In fact, reducing the size of the predicted 5′-UTR sequence (https://fungi.ensembl.org/Sporothrix_brasiliensis_5110_gca_000820605/Gene/Sequence?db=core;g=SPBR_04305;r=Cont16:815228-816386;t=KIH93501) or the length of the 5′-upstream promoter regions of the gene led to a drastic reduction in the expression level. These data indicate the need to evaluate *S. brasiliensis* promoter motifs that account for differential gene expression in future studies.

In this study, we also report the construction and validation of stable fluorescent-tagged *S. brasiliensis* strains and their detection by flow cytometry. By performing a proof-of-principle study, we combined the use of green fluorescent-tagged *S. brasiliensis* and flow cytometry to assess *in vitro* infection of human peripheral blood mononuclear cells (PBMCs) and monocyte-derived macrophages. Our study confirmed that these cells internalize *S. brasiliensis* (8, 29–31). We showed that the sGFP-tagged *S. brasiliensis* strain serves as a study model as its use allowed us to visualize it in phagocytes during infection allowing for flow cytometry-based analysis of this process. The fluorescent-tagged strain, sGFP or mCherry, may represent an ideal tool for an in-depth study with precise time course experiments and a follow-up of the infection by image-based analysis of fluorescence microscopy and flow cytometry. In addition, the ATMT protocol allows the generation of different fluorescent tagged *S. brasiliensis* strains that may be useful for competition assays in virulence studies, as well as between *S. brasiliensis* and *S. schenckii s str* (8, 30).

## MATERIALS AND METHODS

### Ethics statement.

The functional experiments involving cells isolated from the peripheral blood of healthy volunteers at the Hospital of Braga, Portugal, were approved by the Ethics Subcommittee for Life and Health Sciences (SECVS) of the University of Minho, Portugal (no. 014/015). Experiments were conducted according to the principles expressed in the Declaration of Helsinki, and participants provided written informed consent.

### Microorganisms and culture media.

The *S. brasiliensis* ATCC MYA-4823, ATCC MYA-4824, and HUPE 114158 were used for fungal transformation (13, 15, 32); Dr. Leila Lopes-Bezerra kindly provided the last two strains. Whereas *S. brasiliensis* HUPE 114158 was cultured in the brain heart infusion supplemented with 1% glucose (BHI) medium (Biolife, Italy, pH = 7.8), all the other isolates were cultured at 37°C for 72 h in the yeast extract-peptone-dextrose (YPD) medium (pH = 7.8). Clumps of yeast cells were removed by sterile gaze filtration. The A. tumefaciens strains AGL-1, EHA105, and LBA1100 harboring the binary vector pUR5750 were used to evaluate transformation efficiency (33). A. tumefaciens strains were cultured at 26°C in solid LC supplemented with rifampicin (20 μg/mL) and kanamycin (100 μg/mL) for selective purposes (5). Cultures were maintained at 4°C for short-term storage and were routinely subcultured every 4 to 6 weeks. Escherichia coli XL-1-Blue strain grown at 37°C on Luria–Bertani (LB) medium supplemented with kanamycin (50 μg/mL) was used as the host for plasmid amplification and cloning (5).

### Primers and plasmid strategy.

Plasmid DNA extraction, recombinant DNA manipulation, and E. coli and A. tumefaciens transformation procedures were performed as reported elsewhere (34). Primers, plasmids, and inserts were designed using Primer3Web (https://primer3.org/) and SnapGene (https://www.snapgene.com/). All primers used are available in Table S1 and the plasmid construction strategies are described in Text S1. Briefly, a common plasmid backbone was created with the restriction enzyme AvrII, as a single cutter, from the pPTS608 kindly provided by Dr. Bruce S. Klein (6). These constructs allow the cloning of any gene of interest in the C-terminal with either sGFP or mCherry (Text S1). A synthetic fragment containing the open reading frame of H2A from *S. brasiliensis* (SPBR_03511) was used for cloning (idt; https://eu.idtdna.com/). All cloning PCRs were done with high-fidelity polymerase (New England Biolabs, Ipswich, MA) and cloned in the plasmid backbone of interest by *in vivo* assembly technique (35). All plasmids constructed were confirmed by Sanger DNA sequencing (Stab Vida, Portugal). The pPZP201BK::SUR::gpdA::zKat::TrpC, kindly provided by Dr. Augusto Schrank (22), was used to express the far-red fluorescent protein TURBOFP635/Katushka.

### Agrobacterium tumefaciens-mediated transformation protocol.

A. tumefaciens strain cells were cultured at 28°C while shaking (200 rpm) in 10 mL L.C. broth supplemented with antibiotics for 6 h to 10 h. Subsequently, the culture was centrifuged at 4,000 rpm for 6 min, and the pelleted cells were resuspended with induction medium (IM) with 200 μM acetosyringone (AS) to an optic density (OD_660nm_) of 0.3 (11). To acquire virulence, the culture grew overnight at 26°C, 200 rpm, to an OD_660nm_ of 0.6 to 0.8. In parallel, *S. brasiliensis* cells were cultured at 37°C, 200 rpm, for 24 h in 50 mL YPD (pH 7.8), and at the exponential growth phase, yeast cells were isolated through sterile gaze filtration. Cells were centrifuged (4,500 *g* for 5 min), washed with IM, and adjusted to a final concentration of 1 × 10^8^ cells/mL using direct microscopic counts (Neubauer counting chamber procedures). Different ratios of A. tumefaciens and *S. brasiliensis* cells (1:1; 2:1) were mixed to a final volume of 120 μL and spread onto a sterile membrane (Hybond-C and Hybond-N+, 0.45 μm pore, G.E. Healthcare Limited, UK) and placed on solid IM for co-cultivation at 26 or 28°C for different time periods (24 h, 48 h, 72 h, and 96 h). Before incubation, the plates were air-dried in a biological safety cabinet with the lights off for approximately 30 min (5).

Following co-cultivation, membranes were transferred to tubes with a nonselective YPD medium (pH 7.8) containing cefotaxime (200 μg/mL, Formedium, England) and incubated for 6h at 37°C and 200 rpm. These conditions led to growth inhibition of A. tumefaciens and allowed yeast recovery before hygromycin B selection. Yeast cells were recovered by centrifugation and plated on YPD solid medium with hygromycin B (150 μg/mL). Selection plates were incubated at 37°C for 7 to 9 days and monitored for colony-forming ability. ATMT transformation efficiency was compared between three strains of *S. brasiliensis* ATCC MYA-4823, ATCC MYA-4824, and HUPE 114158. For the selection of the best transformation conditions, only the *S. brasiliensis* ATCC MYA-4823 was used at the 2:1 co-cultivation ratio. Because the HUPE 114158 strain did not grow properly in YPD, the BHI medium was used with the same concentration of hygromycin B. For the selective purpose, yeast nitrogen base (YNB) solid medium (0.16% BD Difco YNB, 2% glucose, 0.5% ammonium sulfate, 25 μg/mL of leucine, 25 μg/mL of histidine, 25 μg/mL of methionine, and 25 μg/mL of uracil), supplemented with Chlorimuron ethyl (30 μg/mL, Santa Cruz Biotechnology, USA) was used for selection of *S. brasiliensis* transformed with A. tumefaciens carrying the plasmid described elsewhere (22).

### Gene copy number analysis.

The HPH gene copy number in five *S. brasiliensis* transformants was determined by the standard curve method (Cts plotted against the logarithm of the DNA copy number) (36). To that, the single copy references of the genes actin-β (SPBR_00592) and glyceraldehyde-3-phosphate dehydrogenase (SPBR_04305; GAPDH) were quantified in the genomic DNA of selected transformants. Two wild-type isolates were used as control. Primers used for this analysis are available in Table S1.

### Evaluation of different promoters through sGFP and mCherry expression.

To analyze differences between the activity of GAPDH and H2A promoters along batch growth, six transformants were randomly selected and grew together with *S. brasiliensis* wild-type cells for 3 days at 37°C, 200 rpm in a 100-mL Erlenmeyer flask with 20 mL of YPD (pH 7.8). For selective purposes, Hygromycin B (150 μg/mL) was used whenever needed. The OD_660nm_ was measured every 5 h, and yeast cells were fixed with paraformaldehyde for 15 min at 4°C. A total of 30,000 events were captured using LSRII flow cytometer (BD Biosciences), and singlets' mean fluorescence intensity (MFI) was measured. Wild-type yeast cells were used to set the autofluorescence threshold. For GAPDH promoter analysis, the MFI of transformants expressing the fluorescent protein under the control of different GAPDH promoter size regions spanning from the −1,571 to −1 nt upstream region of the ATG staring codon (Text S1). For this analysis, five randomly selected strains with 1,571, 1,100 bp, 800 bp, and without the UTR region of the pGAPDH promoters were cultured in a selective YPD (pH 7.8) medium. The OD_660nm_ was adjusted to 0.1 and was cultured at 37°C for 24 h to achieve exponential growth. Yeast cells were fixed with paraformaldehyde for 15 min at 4°C, and the flow cytometer captured 30,000 events. Data were acquired on the LSRII flow cytometer (BD Biosciences) with Diva Software and analyzed using FlowJo software (BD Biosciences). Singlet cells were selected after flow cytometry acquisition. Doublet exclusion was performed by plotting the height or width against the area for forward scatter. Therefore, only events with proportionality between height, width, and area were considered as singlets, and used for MFI determination. Wild-type yeast cells in each period were used to determine the autofluorescence threshold.

### Mitotic stability of transformants.

The mitotic stability of the *S. brasiliensis* transformants was determined by analyzing the stability of the green fluorescent protein (sGFP). To achieve that, five randomly selected transformants and wild-type strains were successively cultured without hygromycin B for five periods of 12 h on a YPD medium (pH 7.8) at 37°C and shaking at 200 rpm. At the end of each period, cells were fixed with paraformaldehyde for 15 min at 4°C, and 30,000 events were captured using the LSRII flow cytometer (BD Biosciences). The wild-type MFI was used to determine the MFI autofluorescence threshold and the percentage of population cells that had lost the green fluorescence during generations analyzed under nonselective conditions. In addition, 10 randomly selected transformants were cultured as described above and platted in a solid nonselective medium. From each transformant, 100 colonies were grown on selective medium, containing hygromycin B. Cell growth was evaluated after 7 days at 37°C.

### Fluorescent microscopy.

*S. brasiliensis* transformants were produced through ATMT with the AGL-1 strain harboring several plasmids, which confers the expression of the fluorescence proteins sGFP, mCherry, or TURBOFP635/Katushka in the cytoplasm or nucleus. During exponential growth, yeast and mycelium cells of ATCC MYA-4823 transformants and wild-type were obtained. For posterior analysis, cells were fixed with paraformaldehyde for 15 min at 4°C. Co-localization with Invitrogen DAPI (4[prime],6-diamidino-2-phenylindole) demonstrated fluorescence in the nucleus. To achieve that, cells were stained with 0.1 μg/mL of DAPI for an hour at room temperature while shaking. Fluorescence microscopy analyzes were performed with the Olympus Widefield Upright Microscope BX61 by either bright-field or fluorescence microscopy. All images were captured using 395 nm/509 nm for excitation and emission, respectively, and the exposition time to the laser beam was automatically set. Images were treated in the ImageJ software.

### Isolation of PBMCs and generation of MDMs.

As previously reported, PBMCs and human-derived macrophages (MDMs) were isolated or differentiated (37, 38). Briefly, PBMCs were enriched from whole blood by density gradient using Histopaque-1077 (Sigma-Aldrich, UK). Cells were washed twice in PBS and resuspended in Complete Roswell Park Memorial Institute Medium (cRPMI-1640) culture medium (RPMI 1640 with 2 mM glutamine; Gibco, Thermo Fisher Scientific, USA), 10% human serum (Sigma-Aldrich, USA), 10 U/mL penicillin/streptomycin, and 10 mM HEPES (Thermo Fisher Scientific, the Netherlands). Monocytes (CD14^+^) were isolated from PBMCs by positive selection using magnetically labeled CD14 Microbeads (Miltenyi Biotec). The isolated monocytes were then resuspended in cRPMI medium with 20 ng/mL recombinant human granulocyte-macrophage colony-stimulating factor (GM-CSF, Miltenyi Biotec), and cells were seeded at a concentration of 1 × 10^6^ cells/mL in 24-well and 48-well plates (Corning Inc., South Korea) for 7 days. The culture medium was refreshed on the fourth day of incubation. Before infection, the acquisition of macrophage morphology was confirmed by visualization in a BX61 microscope (Olympus).

### Phagocytosis experiments with green fluorescent tag strains.

Exponential growth yeast cells of pGAPDH::GFP tag-strains were used to infect both PBMCs and MDMs (5 × 10^5^/well in 24-well cell culture plates). Yeast cells were filtered, washed with PBS 1×, and counted using Neubauer chambers with 10-fold dilutions using Trypan Blue (1:10) (Gibco, USA) to consider fungal viability. To achieve an MOI of 1:5 effector-to-target ratio, each well was infected with 20 μL containing 2.5 million yeast cells. The infection was synchronized for 30 min at 4°C, and phagocytosis was initiated by shifting the co-incubation to 37°C at 5% CO2 for 2 h. Phagocytosis was stopped by washing wells with PBS and the percentage of phagocytosis was measured by flow cytometer and fluorescence microscopy. For microscopic analysis, cells were washed with PBS, fixed with 10% formalin for 10 min at room temperature, and the number of internalized fungi inside the PBMCs and macrophages were counted using the Olympus Widefield Upright Microscope BX61. For FACS analysis, cells were washed with PBS and incubated with 250 μL of Accutase, clone:M5E2 (GRiSP, Portugal) at 37°C for 10 min to detach the cells. Cells were then dislodged with a pipette into a 96-well u-shaped cell culture plate and stained with Brilliant Violet 650 anti-human CD14 Antibody (BioLegend, USA) for 10 min at 4°C. Then, the supernatant was removed, and cell were resuspended in 100 μL of PBS, transferred to flow cytometer tubes, and analyzed by flow cytometer. Wild-type *S. brasiliensis* cells had their fungal wall stained with Calcofluor-White (0.1 mg/mL, Sigma-Aldrich, 10 min at RT) and were submitted to the same protocol as previously described to evaluate phagocytosis in PBMC by flow cytometry.

### Cytokine production.

To quantify cytokine production, PBMCs (5 × 10^5^/well in 24-well plates) were infected with exponential growth yeast cells of pGAPDH::GFP tagged strains and wild-type strain at a 1:10 effector-to-target ratio for 24 h, at 37°C and 5% CO2. After stimulation, supernatants were collected and analyzed through Enzyme-Linked Immunosorbent Assay (ELISA), using human TNF, IL-6 e IL-10 ELISA MAX Deluxe Set Kits (BioLegend), according to the manufacturer´s instructions.

### Statistical analysis.

Data are reported as the mean ± standard deviation (SD) of at least two independent assays with three replicates. The statistical analysis was performed using GraphPad Prism Software version 8.0 (GraphPad Software Inc, CA, USA). The normality assumptions were assessed in all cases using the Shapiro-Wilk test. A Student's *t* test was used to analyze the differences in the average number of HygB^R^ clones for co-cultivation temperature and the use of sterile membranes. Differences regarding the time, ratio of co-cultivation, and strains used were analyzed using one-way ANOVA with a Tuckey’s multiple comparation test The MFI differences between promoters in different periods were evaluated using two-way ANOVA and Sidak’s multiple comparation test. The percentage of phagocytosis and cytokine production was compared by either one-way ANOVA or two-way ANOVA. Statistically significant values are indicated as follows: *, *P ≤ *0.05; **, *P ≤ *0.01; ***, *P ≤ *0.001; and ****, *P ≤ *0.0001.
