# Supplementary material for: Development of a Versatile Toolbox for Genetic Manipulation of Sporothrix brasiliensis
Source: Microbiol Spectr. 2023 Feb 27;11(2):e04564-22. doi: 10.1128/spectrum.04564-22 (PMC10101026; doi:10.1128/spectrum.04564-22)
Supplement: Supplemental file 1 — Fig. S1 and S2, Table S1, and Text S1. Download spectrum.04564-22-s0001.pdf, PDF file, 0.8 MB [file spectrum.04564-22-s0001.pdf]

Supplementary Materials

Manuscript:

“**Development of a versatile toolbox for genetic manipulation of *Sporothrix brasiliensis*”**

This file includes:

. Supplementary Figure 1;

. Supplementary Figure 2;

. Tab. S1. Primers List;

. Text S1. Plasmid Construction Strategy.

Supplementary Figure 1

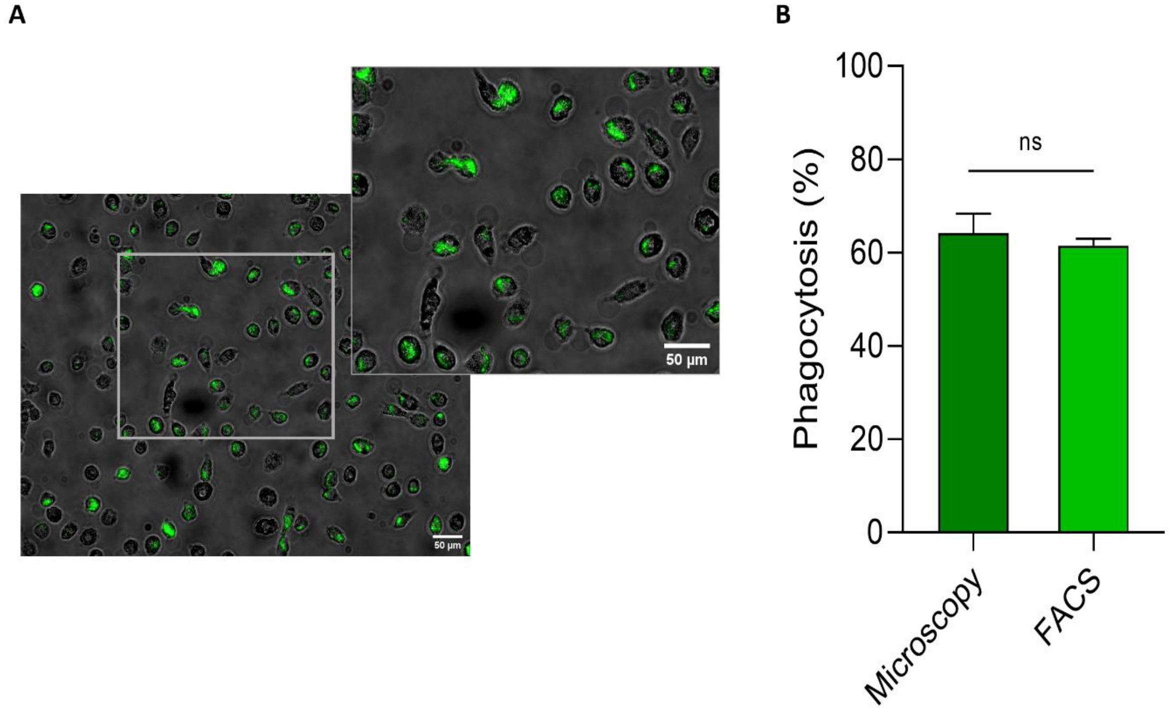

Supplemental Figure. 1: Phagocytosis of MDMs using the green fluorescent *S. brasiliensis* tag strain. The scale bar equals 50µm for both images. **(A)** The green layer and bright fields were merged to visualize the green fluorescence of the *S. brasiliensis* tagged strain inside macrophages. **(B)** Phagocytosis was measured after infection with *S. brasiliensis* sGFP tagged strain cells at the MOI of 1:5 for 2h, using flow cytometry and fluorescence microscopic. Bars depict the percentage of phagocytosis ± SD. No statistical difference was observed ($n \geq 6$).

Supplementary Figure 2

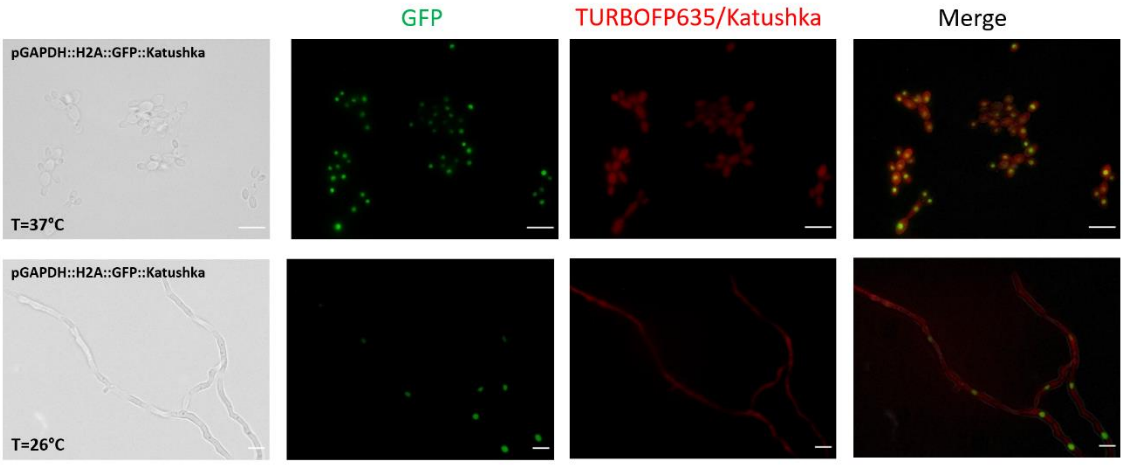

Supplemental Figure. 2: Production of fluorescent *S. brasiliensis* tagged strains. Fluorescence microscopic analysis of H2A::GFP -Katushka *S. brasiliensis* tag strain. During yeast and mycelium phases, green fluorescence is shown in the nucleus and red fluorescence in the cytosol.

. Tab. S1. Primers List

Tab. S1. Primers List

Primer name	SEQUENCE	Observations
HPH_F	ATGCCTGAACTCACCGCGAC	HPH resistance gene in PTS608 plasmids and derivatives
HPH_R	TTCTACACAGCCATCGGTCC	
pLUO_mcherry	CAGAACTCTGTCCAGGTCATGTAATATCACACGCAGCCGAAGCCTTCAG	Plasmid construction: mCherry fragment
H2A-mcherry1	ATGGTGAGCAAGGGCGAGGA	Plasmid construction: pH2A-mCherry and pH2A-H2A-mCherry
H2A-mcherry2	CCATGTTATCCTCCTCGCCC	
H2A-mcherry3	ATGGCTGGCGGCAAGGGAAAAT	
H2A-mcherry4	ATCTGCGGAACATATACTGGGCCCGGGAATTCCGGTGACGTTTGGTGCAG	
H2A-mcherry5	CCGACGATTTTCCCTTGCCGCCAGCCATTGTGTCACGAAAGCGAAGTTG	
H2A-mcherry6	ATGTTATCCTCCTCGCCCTTGCTCACCATTGTGTCACGAAAGCGAAGTTG	
GAPDH_promoter_1	CATCTGCGGAACATATACTGGGCCCGGGAACTCGCTCGTCTTGGAGTGCT	Plasmid construction: promoter GAPDH- mCherry or GFP
GAPDH_promoter_2	CTCCTCGCCCTTGCTCACCATTTTGACTTTGGAATTGAGTTAGCC	
seq_pH2A	GCCTTACTGTATCGCCTCTG	sequencing primers
seq_pGADPH	CCTGGTGACGACTGGCTC	
Linker_GFP	AGTTCTTCTCCTTTACTCATACCAGAGGTGGCGACCGGTG	Plasmid construction: GFP gene and H2A location sequence
H2A_GAPDH	ATTTTCCCTTGCCGCCAGCCATTTTGACTTTGGAATTGAGTTAGCC	
GFP_F	ATGAGTAAAGGAGAAGAACTTTTCAC	
pGAPDH_GFP	AGTTCTTCTCCTTTACTCATTTTGACTTTGGAATTGAGTTAGCC	Plasmid construction: promoter GAPDH with different sizes
GAPDH_1000	GAACATATACTGGGCCCGGGAAGCAGCCCATGACGACCCATT	
GAPDH_800	GAACATATACTGGGCCCGGGAATGCTACCCCGCCATCGATCG	
GAPDH_c/UTR_R	GATCCGTGCTTCCCGATGCCCCTAGGTTGACTTTGGAATTGAGTTAGC	strategy with pGAPDH and linker
GAPDH_s/UTR_R	GATCCGTGCTTCCCGATGCCCCTAGGGAAACTCGAACCGGAG	
RT_LP_actin beta	GATCGGTATGGGCCAGAAGG	Primers list to copy number analysis (RT-PCR)
RT_RP_actin beta	GGATACCACGCTTCGACTGT	
RT_LP_GAPDH	TGCCTCCTACGACGAGATCA	
RT_RP_GAPDH	GTGTAGCCGAGAATGCCCTT	
RT_LP_HPH	GATGTAGGAGGGCGTGGATA	
RT_RP_HPH	ATAGGTCAGGCTCTCGCTGA	

Text S1. Plasmid Construction Strategy;

Promoter's strategy

Utilizing the fungi.ensembl.org database, we located the sequences referring to the histone H2A (SPBR_03511) and glyceraldehyde 3-phosphate dehydrogenase (SPBR_04305) genes. The respective first 600 bp upstream of the exonic region presented in the ORF sequence was considered the 5'UTR region. After that, 893 bp and 971 bp upstream of the 5'UTR region were respectively selected, and the total sequence was used in the construction of the respective promoter. The GAPDH division was performed using the first 600 bp as the 5'UTR region of the GAPDH promoter. Upstream the 5'UTR region, a fragment with 157 bp was maintained in all constructions with this promoter and considered the core region. The following fragment from the core upstream region was considered the UAS region.

Plasmids pH2A-mCherry and pGAPDH-mCherry

The pPTS608-Cas9-HygB backbone vector was cut with the enzymes BglII (New England Biolabs®) and PmlI (New England Biolabs®), and two fragments were inserted. For the plasmid with pH2A-mCherry – The first used primers H2A-mcherry4 and H2A-mcherry6 to amplify the H2A promoter from the gDNA of *S. brasiliensis*. The second used primers pLUO_mcherry and H2A-mcherry1 to amplify the mCherry. For the plasmid with pGAPDH-mCherry, we used GAPDH_promoter_1 and GAPDH_promoter_2 primers to amplify the GAPDH promoter from the *S. brasiliensis* gDNA, and the primers cas9_mcherry 4 and H2A-mcherry1 to amplify the mCherry.

Plasmids pH2A_H2A-mCherry and pGAPDH_H2A-GFP

The pPTS608-Cas9-HygB backbone vector was cut with the enzymes BglII (New England Biolabs®) and PmlI (New England Biolabs®), and three fragments were inserted.

pH2A_H2A-mCherry – (1) used primers H2A-mcherry4 and H2A-mcherry5 to amplify the H2A promoter from *S. brasiliensis* gDNA. (2) used primers H2A-mcherry2 and H2A-mcherry3, the coding region of the H2A gene, a linker sequence, and the initial part of the mCherry fluorescence protein gene. (3) Primer cas9_mcherry 4 and H2A-mcherry1 were used to amplify the mCherry.

pGAPDH_H2A-GFP – (1) GAPDH promoter amplified from gDNA *S. brasiliensis* (primers GAPDH_promoter_1 and H2A_GAPDH). (2) target sequence H2A (primers H2A-mcherry3 and Linker_GFP). (3) GFP gene (primers cas9_mcherry 4 and GFP_F).

Plasmids GFP and Promoter GAPDH with different sizes

The plasmids were designed: pPTS608_pGAPDH-GFP, pPTS608_p1157GAPDH-GFP, pPTS608_p757GAPDH-GFP, and pPTS608_pGAPDH-without_5'UTR-GFP. The pPTS608-Cas9-HygB plasmid was digested with the enzymes BglII and PmlI, and two fragments were inserted. The first used gDNA from *S. brasiliensis* to amplify the GAPDH promoter, and promoter primers vary depending on the size of the desired promoter. For the complete GAPDH promoter primers GAPDH_promoter_1 and pGAPDH_GFP, the 1157 bp primers GAPDH_1000 and

pGAPDH_GFP, and the 757 bp promoter primers GAPDH_800 and pGAPDH_GFP. The second remains the same across all plasmids and is designed to amplify the GFP gene with primers pLUO_mcherry and GFP_F. For the construction of pPTS608_pGAPDH-without_5'UTR-GFP, a different strategy was used; the Plasmid pPST608_GAPDHfull_H2A_GFP was used as a vector backbone and digested with ApaI and XhoI and the promoter to be inserted made by amplifying the *S. brasiliensis* genomic gDNA with the primers GAPDH_promoter_1 and GAPDH_s/UTR_R.

pPTS608_pGAPDH-linker-GFP and pPTS608_pGAPDH-linker-mCherry

For the design of plasmids that express fusions of fluorescence proteins with a specific region of the fungal cell, new plasmids with the fluorescence genes GFP and mCherry were produced. The plasmids pPTS608_pH2A_H2A-mCherry and pPTS608_pGAPDH_H2A-GFP were used as a backbone vector. They were digested with ApaI (Thermo Fisher Scientific) and XhoI (Thermo Fisher Scientific) enzymes. After digestion, an amplified fragment of *S. brasiliensis* gDNA containing the desired promoter is inserted. These plasmids retain the original vector linker in addition to receiving the AvrII cleavage site, allowing easy editing and insertion of the desired location sequence. Its backbone vector is pPTS608_pGAPDH_H2A-GFP or pPTS608_pH2A_H2A-mCherry, which is digested as above. These are plasmids without localization sequences containing linker, GFP or mCherry gene sequence, and AvrII enzyme digestion site. Its promoter is amplified using primers GAPDH_promoter_1 and GAPDH_c/UTR_R in *S. brasiliensis* gDNA,
